# 1353. Gonorrhea and Chlamydia Testing and Case Rates Among Women Veterans in the Veterans Health Administration

**DOI:** 10.1093/ofid/ofab466.1545

**Published:** 2021-12-04

**Authors:** Shimrit Keddem, Marissa Maier, Carolyn Gardella, Joleen Borgerding, Elliott Lowy, Maggie Chartier, Sally Haskell, Ronald Hauser, Lauren Beste

**Affiliations:** 1 Department of Veterans Affairs, Philadelphia, Pennsylvania; 2 VA Portland Health Care System/Oregon Health and Sciences University, Portland, OR; 3 University of Washington/ Veterans Health Administration, Seattle, Washington; 4 VA Puget Sound Health Care System, Seattle, Washington; 5 VA Puget Sound HCS, Seattle, Washington; 6 San Francisco VA Medical Center, San Francisco, CA; 7 US Department of Veterans Affairs, New Haven, Connecticut; 8 Yale University School of Medicine, West Haven, Connecticut

## Abstract

**Background:**

United States (US) rates of sexually transmitted infection (STI) in women, especially gonorrhea and chlamydia, have increased over the past decade. Women Veterans have many risk factors associated with STIs, including high rates of childhood sexual assault, military sexual trauma and intimate partner violence. Despite the availability of effective diagnostic tests and evidence-based guidelines for annual screening among sexually active women under age 25, screening rates for gonorrhea and chlamydia remain low in the US and among Veterans.

**Methods:**

We performed a retrospective cohort study of all women Veterans in Veterans Health Administration (VHA) care between January 1, 2018 and December 31, 2019 to examine patient characteristics and health system factors associated with gonorrhea and chlamydia testing and case rates among women Veterans in the VHA in 2019.

**Results:**

Among women under age 25, 21.3% were tested for gonorrhea or chlamydia in 2019. After adjusting for demographic and other health factors, predictors of testing in women under age 25 included Black race (aOR: 2.11 CI: 1.89, 2.36), rural residence (aOR: 0.84, CI: 0.74, 0.95), and cervical cancer screening (aOR: 5.05 CI: 4.59, 5.56). Women under age 25 had the highest infection rates, with an incidence of chlamydia and gonorrhea of 1,950 and 267 cases/100,000, respectively. Incidence of gonorrhea and chlamydia was higher for women with a history of military sexual trauma (MST) (Chlamydia case rate: 265, Gonorrhea case rate: 97/100,000) and those with mental health diagnoses (Chlamydia case rate: 263, Gonorrhea case rate: 72/100,000.) Over a third of chlamydia cases (35.2%) and gonorrhea cases (35.5%) occurred in women who resided in the South Atlantic census division.

Chlamydia cases per 100,000 women Veterans seen in VHA (2019)

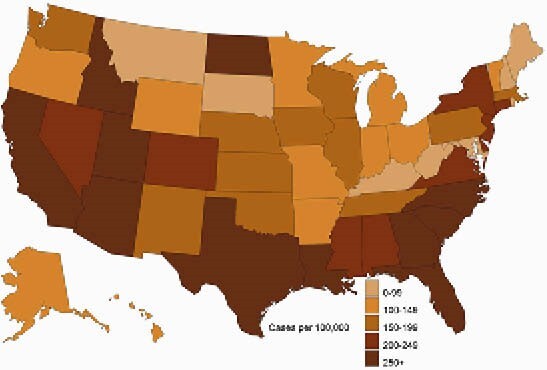

Gonorrhea cases per 100,000 women Veterans seen in VHA (2019)

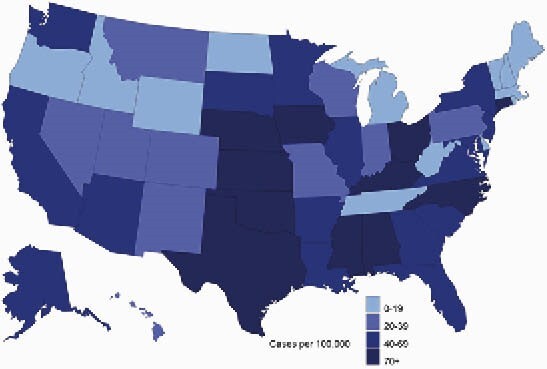

**Conclusion:**

Gonorrhea and chlamydia testing remains underutilized among women in the VHA and infection rates are high among younger women. Patient-centered, system-level interventions are urgently needed to address low testing rates.

**Disclosures:**

**All Authors**: No reported disclosures

